# Construction of Electrostatic Self-Assembled 2D/2D CdIn_2_S_4_/g-C_3_N_4_ Heterojunctions for Efficient Visible-Light-Responsive Molecular Oxygen Activation

**DOI:** 10.3390/nano11092342

**Published:** 2021-09-09

**Authors:** Hongfei Yin, Chunyu Yuan, Huijun Lv, Xulin He, Cheng Liao, Xiaoheng Liu, Yongzheng Zhang

**Affiliations:** 1 School of Physics and Physical Engineering, Qufu Normal University, Qufu 273165, China; yinhongfei1016@foxmail.com (H.Y.); chunyuyuan.qfnu@gmail.com (C.Y.); hjlv.qfnu@gmail.com (H.L.); 2 Chengdu Science and Technology Development Center, China Academy of Engineering Physics, Chengdu 610000, China; hexl0003@yinhe596.cn (X.H.); cliao@pku.edu.cn (C.L.); 3 Key Laboratory for Soft Chemistry and Functional Materials of Ministry of Education, School of Chemical Engineering, Nanjing University of Science and Technology, Nanjing 210094, China

**Keywords:** 2D/2D, electrostatic self-assembled, heterojunction, photocatalytic, molecular oxygen activation

## Abstract

Molecular oxygen activated by visible light to generate radicals with high oxidation ability exhibits great potential in environmental remediation The efficacy of molecular oxygen activation mainly depends on the separation and migration efficiency of the photoinduced charge carriers. In this work, 2D/2D CdIn_2_S_4_/g-C_3_N_4_ heterojunctions with different weight ratios were successfully fabricated by a simple electrostatic self-assembled route. The optimized sample with a weight ratio of 5:2 between CdIn_2_S_4_ and g-C_3_N_4_ showed the highest photocatalytic activity for tetracycline hydrochloride (TCH) degradation, which also displayed good photostability. The enhancement of the photocatalytic performance could be ascribed to the 2D/2D heterostructure; this unique 2D/2D structure could promote the separation and migration of the photoinduced charge carriers, which was beneficial for molecular oxygen activation, leading to an enhancement in photocatalytic activity. This work may possibly provide a scalable way for molecular oxygen activation in photocatalysis.

## 1. Introduction

The energy crisis and environmental pollution are serious problems worldwide. Environmental pollution originating from refractory organic pollutants, especially antibiotics, has an extremely negative influence on humans. Photocatalytic technology has been recognized as a potential way to mitigate environmental pollution because titanium dioxide is used as the catalyst for water splitting under UV light irradiation [1]. However, the relatively large bandgap of TiO_2_ renders it unacceptable in handling the above-mentioned environmental problems with high efficiency. Therefore, it is strongly desirable that photocatalysts with high solar utilization be explored [2,3,4,5,6,7,8].

CdIn_2_S_4_, a ternary sulfide of chalcogenide, with an appropriate bandgap and suitable band edge positions, has gained increasing attention in the scope of photocatalysis, due to its potential applications in photocatalytic hydrogen production [9,10,11,12], organic conversions [13,14], and organic pollutant degradation [15,16,17], as well as CO_2_ reduction [18,19]. However, two issues have greatly restricted the widespread use of pure CdIn_2_S_4_. One is the fast recombination of photogenerated charge carriers, and the other is photo-corrosion. During the photocatalytic process, S^2−^ can be oxidized by photoinduced holes [20], and the generated dissociative Cd^2+^ would have a negative influence on living organisms. Therefore, it is desirable to design an effective CdIn_2_S_4_-based photocatalyst without sacrificing photocatalytic performance and using less Cd source. It was reported that doping heteroatoms [21] or constructing heterojunctions [22,23,24] were efficient methods of alleviating the two above-mentioned problems, where the construction of heterojunctions could inhibit the speedy recombination of photoexcited charge carriers and alleviate the photo-corrosion more efficiently, owing to the spatial location of photoexcited electrons and holes.

In recent decades, owing to the properties of earth abundance, non-toxicity, a simple preparation process and stable structure, g-C_3_N_4_, which belongs to a type of metal-free photocatalyst, has been widely used for building heterojunctions [25,26,27,28,29,30]. Nevertheless, bulk g-C_3_N_4_ exhibits low specific surface areas and suffers from the rapid recombination of photoinduced electron-hole pairs; these drawbacks seriously limit photocatalytic efficiency. Exfoliating bulk g-C_3_N_4_ into two-dimensional nanosheets with few layers or single layers is an effective method for improving the photocatalytic activities of g-C_3_N_4_ [31,32,33]. Wang et al. described an ultrasonic exfoliation route for fabricating g-C_3_N_4_ nanosheets with boosted photocatalytic performance; the abundant reaction active sites and the low recombination rate of charge carriers were attributed to the enhanced photocatalytic performances [34]. Qu et al. combined freeze-dried, ultrasonic and solvothermal process-synthesized g-C_3_N_4_ nanosheets with an atomically thin mesoporous structure that exhibited superior photocatalytic hydrogen evolution performance; the ultrathin nanostructure could promote light absorption as well as shorten the migration time and migration distance of photoexcited charge carriers [35].

It has been reported that the 2D/2D nanostructures have tight interfacial contact and a large contact area, which not only provides more channels for carrier transfer, but also shortens the transfer time and migration distance, leading to improved photocatalytic performance [36,37,38,39,40,41]. It is expected that coupling 2D CdIn_2_S_4_ with 2D g-C_3_N_4_ is an efficient method to enhance the photocatalytic activities of CdIn_2_S_4_ while using less of the Cd species. In this work, 2D/2D CdIn_2_S_4_/g-C_3_N_4_ nanocomposites with various weight ratios were constructed through a simple and low-cost electrostatic self-assembled method. Various characterization technologies were utilized to fully study the crystallization, morphology, optical and electrochemical properties of the obtained 2D/2D CdIn_2_S_4_/g-C_3_N_4_ heterojunctions. The photocatalytic performance of the obtained 2D/2D CdIn_2_S_4_/g-C_3_N_4_ heterojunctions was estimated through TCH degradation under visible light illumination. The constructed 2D/2D nanostructures could efficiently facilitate the separation and transfer of photoexcited charge carriers between hetero-interfaces, which is favorable for the process of molecular oxygen activation, resulting in improved photocatalytic activity.

## 2. Materials and Methods

### 2.1. Reagents

Urea (AR) and indium chloride (InCl_3_•4H_2_O, AR) were bought from Sigma Aldrich (Shanghai, China). Cadmium acetate [Cd (CH_3_COO)_2_·2H_2_O, AR] and thioacetamide (C_2_H_5_N_S_, AR) were provided by Sinopharm Chemical Reagent Co. Ltd. (Shanghai, China). All the chemical reagents were used without further treatment.

### 2.2. Synthesis of the Photocatalysts

#### 2.2.1. Synthesis of g-C_3_N_4_ Nanosheets and Protonated g-C_3_N_4_ Nanosheets

First, bulk g-C_3_N_4_ was fabricated through thermal condensation of urea. Typically, 20 g urea was set in a covered crucible, then heated to 550 °C within 240 min and maintained at this temperature for 240 min to obtain bulk g-C_3_N_4_, denoted as BCN. g-C_3_N_4_ nanosheets were obtained via a secondary calcination of the BCN with the same calcination procedure for BCN, and the generated samples were ground for further use and denoted as CNNSs.

Protonated g-C_3_N_4_ nanosheets were prepared on the basis of previous reports with some modifications [42,43]. Typically, 2 g CNNSs were added to 300 mL 1M HCl aqueous solutions; after ultrasonic treatment for 1 h, a homogeneous suspension was formed, which was further stirred for 4 h to promote the protonation process, the protonated g-C_3_N_4_ nanosheets were obtained via centrifugation, followed by washing with a large amount of distilled water to eliminate the excess HCl until pH = 7. Finally, the protonated g-C_3_N_4_ nanosheets were dried at 60 °C overnight and denoted as PCNNSs.

#### 2.2.2. Preparation of CdIn_2_S_4_ Nanosheets

The CdIn_2_S_4_ nanosheets were prepared as described previously with some adjustment [44]. In a typical preparation procedure, 399 mg Cd (CH_3_COO)_2_·2H_2_O and 880 mg InCl_3_•4H_2_O were added into 300 mL deionized water. After 30 min stirring at room temperature, 600 mg thioacetamide (TAA) was added. Then, the temperature was raised to 100 °C from room temperature after 30 min stirring, and the reaction system was refluxed at this temperature for 12 h under continuous magnetic stirring. After the reaction was finished, the produced samples were collected by centrifugation and then cleaned with deionized water 2 times. Finally, after drying at 60 °C overnight, the final product was denoted as CIS.

#### 2.2.3. Synthesis of 2D/2D CdIn_2_S_4_/g-C_3_N_4_ Heterojunctions

CdIn_2_S_4_ nanosheets and PCNNSs were simultaneously dispersed into deionized water with the assistance of ultrasonic treatment for 2 h to form 2 uniform suspensions with a concentration of 0.75 mg/mL. Then, the PCNNSs dispersion was dropwise added into the CdIn_2_S_4_ nanosheet dispersion under continuous magnetic stirring at room temperature; after 6 h, the mixed suspension was separated by centrifugation, then dried at 60 °C in a vacuum oven overnight. The final mass ratios of CdIn_2_S_4_ to PCNNSs were 5:1, 5:2, 5:3, and 5:4, denoted as CISCN-1, CISCN-2, CISCN-3, and CISCN-4, respectively.

### 2.3. Characterization

The crystal nature of the obtained CdIn_2_S_4_/g-C_3_N_4_ hybrids was examined by powder X-ray diffraction (XRD) performed on a Bruker D8 Advance instrument. The morphology of the fabricated samples was observed by field-emission scanning electron microscope (FESEM, Hitachi S-4800) and transmission electron microscope (TEM, JEOL JEM-2100). The optical properties of the as-prepared samples were measured by ultraviolet-visible (UV-vis) diffuse reflection spectra (DRS) using a Shimadzu UV-3100 spectrophotometer, where BaSO_4_ was used as reference, with a test range of 200−800 nm. Element composition and chemical state of each element on the surface of the as-prepared photocatalysts were detected on a PHI Quantera II SXM photoelectron spectrometer under Al Kα radiation (λ = 0.84 nm).

### 2.4. Electrochemical Analysis

The photoelectrochemical tests, including transit photocurrent response and electrochemical impedance spectra (EIS), were characterized by a three-electrode electrochemical system on a CHI760E electrochemical workstation. In the test system, Ag/AgCl electrode and Pt wire electrode were utilized as reference electrode and counter electrode, respectively. A 300 W Xe lamp was employed to provide visible light illumination. For transit photocurrent measurement, the electrolyte was Na_2_SO_4_ aqueous solution with a concentration of 0.5 M (pH ≈ 6.8). For EIS measurements, the electrolyte was a mixed solution containing 0.5 M KCl and 5 mM K_3_[Fe(CN)_6_]/K_4_[Fe(CN)_6_], and the signals were recorded from 100 kHz to 0.01 Hz, respectively. The working electrode was fabricated as follows: 4 mg of the obtained catalysts were dispersed into a mixture solvent of 750 μL water and 250 μL ethanol solution. Then, 10 μL Nafion solution (5 wt%) was added. The mixture was ultrasonicated for 60 min to form a homogeneous slurry. Finally, 100 μL of the dispersions were loaded onto a 1 cm × 3 cm ITO-coated glass substrate with coating area of nearly 1 cm^2^.

### 2.5. Catalytic Experiments

The photocatalytic performance of the fabricated samples was evaluated by TCH degradation under visible light illumination. A 300 W Xe lamp attached with a 400 nm cut-off filter was used to provide visible light. Typically, 30 mg sample was dispersed into 50 mL 50 mg/L TCH aqueous solution. Then, the mixture was stirred for 1 h in darkness to promote adsorption–desorption equilibrium between the sample and the TCH. After the reaction system was exposed to visible light, 3 mL suspension was sucked out every 20 min. After removing the catalyst from the suspension, the remained filtrate was analyzed to determine the concentration of TCH.

### 2.6. Quantitative Analysis of •O_2_^−^

The generation of •O_2_^−^ was measured by nitroblue tetrazolium (NBT) conversion strategy. Due to the reaction between •O_2_^−^ and NBT at a mole ratio of 4:1, the concentration of •O_2_^−^ could be determined by the decrease in NBT. In a typical NBT transformation reaction procedure, 10 mg sample was added into 50 mL 0.05 mM NBT aqueous solution. Then, the mixed solution was stirred continuously in the dark for 60 min to promote the adsorption–desorption equilibrium between the sample and the NBT. After the reaction system was exposed to visible light, 3 mL solution was sucked out every 10 min. After removing the catalyst via a millipore filter (0.22 μm), UV-vis spectrometer (UV-1801) was used to test the concentration of NBT.

## 3. Results and Discussion

**Figure 1:**
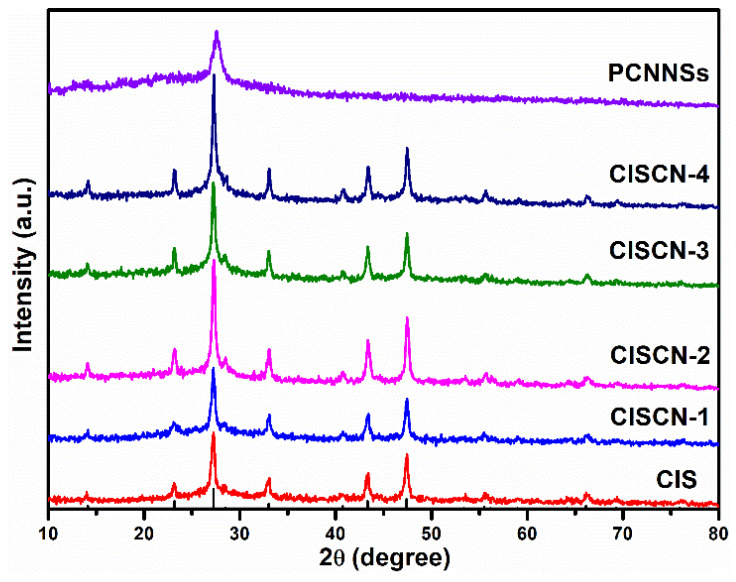
XRD patterns of the fabricated catalysts.

The crystalline structure of the as-fabricated CdIn_2_S_4_/g-C_3_N_4_ heterojunctions and single component was studied by powder XRD, as displayed in Figure 1. The apparent diffraction peak at 2θ = 27.6° in PCNNSs could be assigned to the (002) planes of graphitic materials, which represent the interlayer stacking of a conjugated aromatic structure [45]. For CdIn_2_S_4_, the diffraction peaks of 2θ at 14.1°, 23.2°, 27.2°, 28.5°, 33.0°, 40.7°, 43.3°, 47.4°, 55.5°, and 66.1° could be indexed into (111), (220), (311), (222), (400), (422), (511), (440), (533), and (731) crystal planes of CdIn_2_S_4_ (JCPDS NO.27-0060) with cubic phase structure. All the diffraction peaks of CdIn_2_S_4_/g-C_3_N_4_ heterojunctions were similar to those of pure CIS, which indicated the existence of CIS in the CISCN heterojunctions. However, the diffraction peak of PCNNSs could not be clearly observed in the CdIn_2_S_4_/g-C_3_N_4_ nanocomposites, which might have originated from a lower peak intensity than that of CIS in the range of 27.2° and 28.5°.

**Figure 2:**
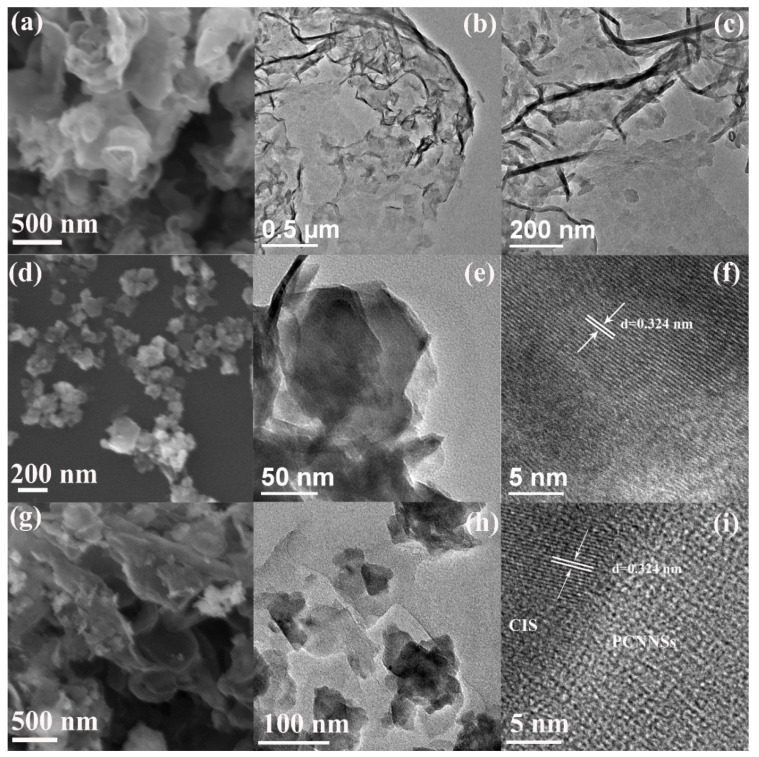
SEM of (**a**) PCNNSs, (**d**) CdIn_2_S_4_, (**g**) CISCN-2, TEM of (**b**,**c**) PCNNSs, (**e**) CdIn_2_S_4_, (**h**) CISCN-2 and HRTEM of (**f**) CdIn_2_S_4_, (**i**) CISCN-2.

The morphologies of the as-prepared PCNNSs, CIS, and CISCN-2 were observed via FESEM and TEM, as shown in Figure 2. The characteristic SEM pattern of the PCNNSs is displayed in Figure 2a; it exhibited a nanostructure of nanosheet, which was further verified by the TEM test, as depicted in Figure 2b,c. Figure 2d shows the SEM image of CIS, which displayed a small nanosheet-like morphology with a size of about 100–200 nm, in accordance with the TEM result (Figure 2e). The high-resolution TEM (HRTEM) pattern of the CIS is illustrated in Figure 2f; the observable lattice space of 0.324 nm marked in Figure 2f could be indexed to the (311) crystal plane of CdIn_2_S_4_. As for the CISCN-2 nanocomposite, from SEM observations (Figure 2g), it displayed a morphology similar to that of PCNNSs, which might be due to the smaller size of the CIS nanosheets. TEM (Figure 2h) and HRTEM (Figure 2i) were used to further investigate its nanostructure, Low-resolution TEM (Figure 2h) revealed that the small CIS nanosheets were stacked on the surface of the PCNNSs nanosheets, exhibiting a sheet-on-sheet morphology. Meanwhile, an obvious interface between CIS and PCNNSs could be observed (Figure 2i), implying the CISCN nanocomposite was successfully prepared by the facile electrostatic self-assembled method.

**Figure 3:**
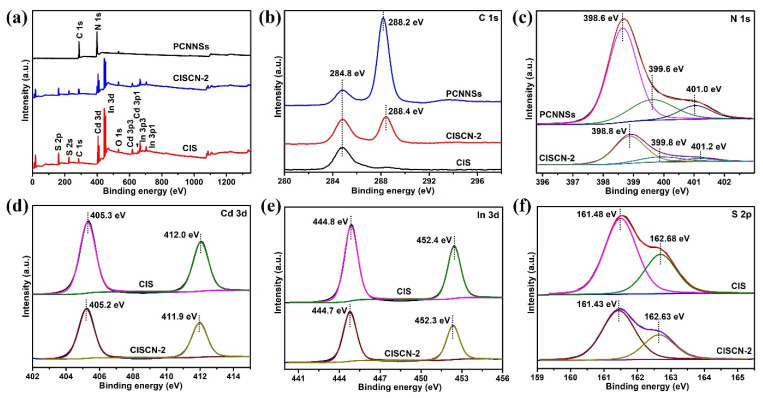
XPS spectrum of PCNNSs, CIS, and CISCN-2: (**a**) full-range spectrum, high resolution XPS spectra of (**b**) C1s, (**c**) N 1s, (**d**) Cd 3d, (**e**) In 3d, and (**f**) S 2p.

The composition of surface elements and the chemical state of each element in the obtained photocatalysts were investigated by XPS survey, as shown in Figure 3. It can be seen from Figure 3a that there were characteristic peaks of C, N, Cd, In, and S in the full spectrum of the as-fabricated samples, implying the as-obtained CISCN-2 heterojunction consisted of CdIn_2_S_4_ and g-C_3_N_4_. The signals of C 1s in PCNNSs were located at 284.8 and 288.2 eV, while the peaks of C 1s in CISCN-2 were located at 284.8 and 288.4 eV (Figure 3b); the former peak could be attributed to the adventitious carbon, while the latter peak could be assigned to N=C-N type carbons [46,47]. As for N 1s (Figure 3c), the characteristic signal in PCNNSs could be divided into three peaks: 398.6 eV (C-N=C), 399.6 eV (N-(C)_3_), and 401.0 eV (N-H) [18,48], while in CISCN-2, these three peaks had a small shift toward higher binding energy, located at 398.8, 399.8, and 401.2 eV, respectively. For Cd 3d (Figure 3d), two obvious peaks at 405.3 and 412.0 eV could be observed in pure CIS, while in CISCN-2, these two peaks exhibited a small shift toward lower binding energy at 405.2 and 411.9 eV, which corresponded to the Cd 3d_5/2_ peak and 3d_3/2_ peak, respectively [49]. Meanwhile, this phenomenon also occurred in the case of In 3d (Figure 3e) and S 2p (Figure 3f); compared with the neat CdIn_2_S_4_, the characteristic peaks of In 3d and S 2p in CISCN-2 also exhibited a small shift toward lower binding energy, indicating the change of chemical environment. This might have originated from the bonding interaction between PCNNSs and CIS.

**Figure 4:**
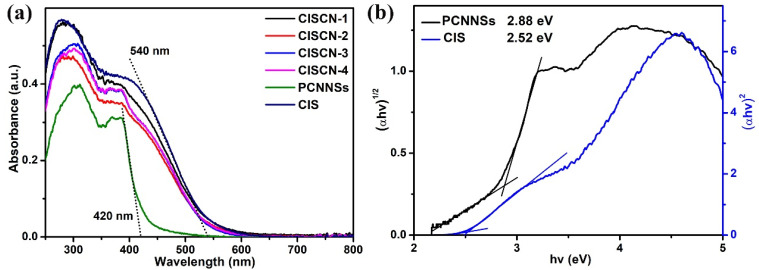
(**a**) UV-vis DRS of the fabricated samples and (**b**) calculation of PCNNSs and CIS bandgap energies.

The optical properties of the attained catalysts were investigated via UV-vis DRS spectra, as displayed in Figure 4a. It can be clearly seen that CIS possessed a higher UV-visible light absorption than pure PCNNSs. The absorption edges of CIS and PCNNSs were about 540 nm and 420 nm, respectively. Compared to the neat CIS, the light absorption over the obtained CdIn_2_S_4_/g-C_3_N_4_ heterojunctions exhibited an obvious decrease, indicating that the introduction of PCNNSs was not helpful for light absorption, which may have been due to the microstructural changes. Therefore, the light absorption might not be responsible for the improved photocatalytic performance. However, the introduction of PCNNSs had a positive influence on the formation of a heterogeneous interface between CIS and PCNNSs; the formation of the heterogeneous interface could promote the transfer of photoinduced electrons and holes [37,38]. The bandgap energies of PCNNSs and CIS were calculated on the basis of the Kubelka–Munk equation and estimated to be 2.88 and 2.52 eV, respectively [50]. The relatively higher bandgap energy of PCNNSs compared to bulk g-C_3_N_4_ may possibly originate from the quantum confinement effect [51].

**Figure 5:**
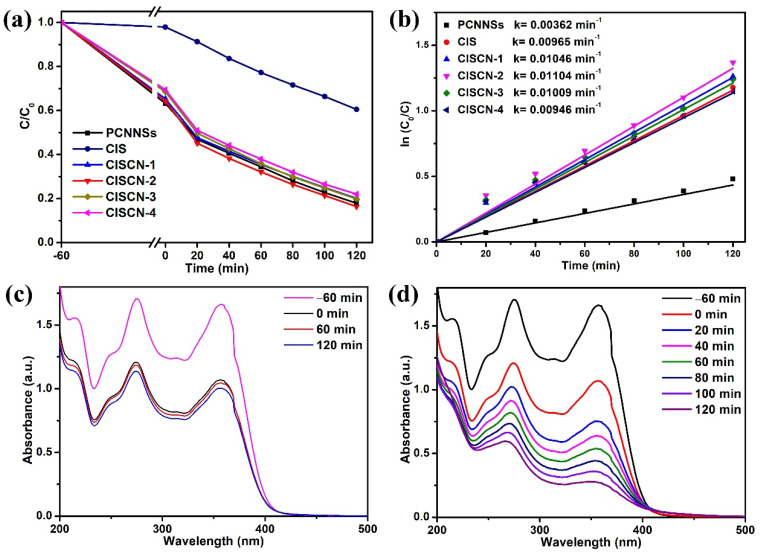
(**a**) Photocatalytic performance of various samples toward TCH photodegradation under visible light illumination; (**b**) the kinetic curves fitted by pseudo-first-order equations over different samples; (**c**) UV-vis spectra of TCH adsorbed by CISCN-2 for different time periods; and (**d**) UV-vis spectra of TCH at different periods of photocatalytic process over CISCN-2.

The photocatalytic performance of the attained photocatalysts was assessed via photocatalytic TCH degradation under visible light irradiation, as illustrated in Figure 5. It can be clearly observed from Figure 5a that pure PCNNSs exhibited the poorest photocatalytic performance among the as-prepared photocatalysts; it had a TCH removal ratio of only 39.5%, whereas pure CIS exhibited a removal ratio of 82.1%. After the formation of CISCN heterojunctions, the removal efficiency of TCH over the constructed CISCN-1, CISCN-2, CISCN-3, and CISCN-4 was 79.9%, 83.6%, 80.1%, and 78.0%, respectively. For comparison, the contribution of adsorption over PCNNSs, CIS, CISCN-1, CISCN-2, CISCN-3, and CISCN-4 was 36.8%, 2.1%, 34.7%, 35.7%, 31.5%, and 30.5%, respectively. Obviously, with the increased content of PCNNSs, the photocatalytic performance of the CISCN nanocomposites tended to increase first and then decrease. This might have originated from the excess amount of PCNNSs, which would lead to an insufficient interface formed between CISCN nanocomposites and PCNNSs and may exhibit a negative effect on the interaction between CIS and PCNNSs, inhibiting the separation of the photoexcited electron-hole pairs. The kinetic reaction procedure of TCH degradation was fitted by pseudo-first-order equations, as shown in Figure 5b. Compared to the single component, the apparent kinetic constant of CISCN-2 was 1.14 and 3.05 times as high as that of the CdIn_2_S_4_ and protonated g-C_3_N_4_, respectively, indicating the superior photocatalytic performance of the CdIn_2_S_4_/g-C_3_N_4_ heterojunction. To verify that the decrease in TCH was triggered by photocatalysis during visible light irradiation, a prolonged adsorption experiment with TCH over CISCN-2 under dark conditions was carried out, as shown in Figure 5c. By prolonging the adsorption time, a delayed decrease could be observed, due to the adsorption–desorption equilibrium established between the TCH and the photocatalyst. Figure 5d shows the UV-vis spectra of TCH at different periods of the photocatalytic process over CISCN-2; the absorbance of TCH had an obvious decrease after 2 h of visible light illumination, implying photocatalysis played an important role in the degradation of TCH. Therefore, the decrease in TCH could be attributed to the collaboration of adsorption and photocatalysis, and during the period of visible light irradiation, the decrease in TCH could be attributed to photocatalysis. To highlight the superiority of the fabricated catalyst, a comparison with previous reports is presented in Table 1.

**Figure 6:**
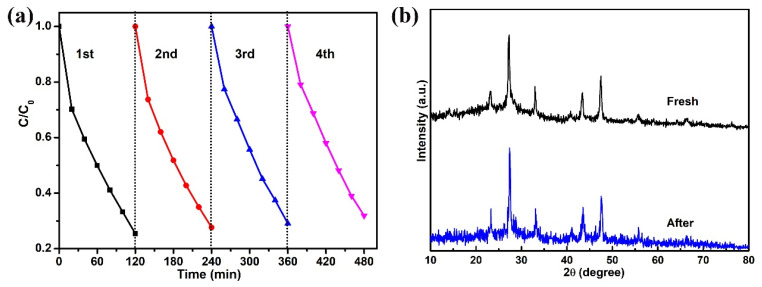
(**a**) Four cyclic experiments of TCH photodegradation over CISCN-2. (**b**) XRD patterns of CISCN-2 before and after cyclic experiments.

The recycling ability of the photocatalyst is a vital factor to evaluate the performance of the photocatalyst; therefore, the progress of the recycling photocatalytic experiment is of great necessity. After each recycling run, the photocatalyst was collected and washed for the next run. It can be observed from Figure 6a that after four recycling runs, the TCH removal ratio over CISCN-2 was still 68.2%; compared to its the first-time usage, there was only an 8.7% reduction, implying the relative stability of the as-prepared CISCN-2. Moreover, XRD of the reused photocatalyst was tested to further confirm the stability of the CISCN-2 composites, as depicted in Figure 6b. Notably, there were no obvious changes in the reused sample as compared to the fresh photocatalyst, suggesting the stability of the crystal structure.

**Figure 7:**
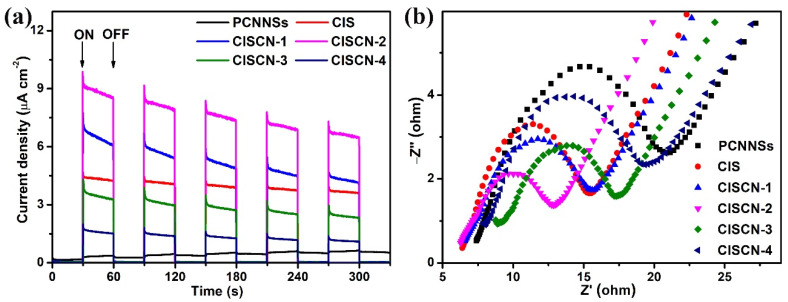
(**a**) Transient photocurrent response plots, and (**b**) EIS of the obtained catalysts.

Generally, three steps are involved in the photocatalytic process: (1) semiconductor photocatalysts were irradiated by light, leading to the formation of photogenerated electrons and holes; (2) photoinduced electrons and holes were separated and transferred to the surface of the photocatalyst; (3) the photoexcited electrons and holes participated in the surface redox reactions [51]. The separation and transfer efficiency of the photoinduced charge carriers are important factors that affect the photocatalytic performance. Transient photocurrent (Figure 7a) was measured to evaluate the separation efficacy of the photoexcited charge carriers, while EIS (Figure 7b) measurement was carried out to estimate the transfer efficiency of the photogenerated electron-hole pairs [52,53]. It can be easily observed from Figure 7a that CISCN-2 displayed the highest photocurrent intensity among all the photocatalysts, implying the highest separation efficacy of the photoexcited electron-hole pairs. Meanwhile, the smallest semicircle of the EIS curve could be observed over CISCN-2, implying the smallest transfer resistance of photoinduced charge carriers and highly interfacial transfer efficacy of the charge carriers. These two results might be reasons for the enhanced photocatalytic performance.

**Figure 8:**
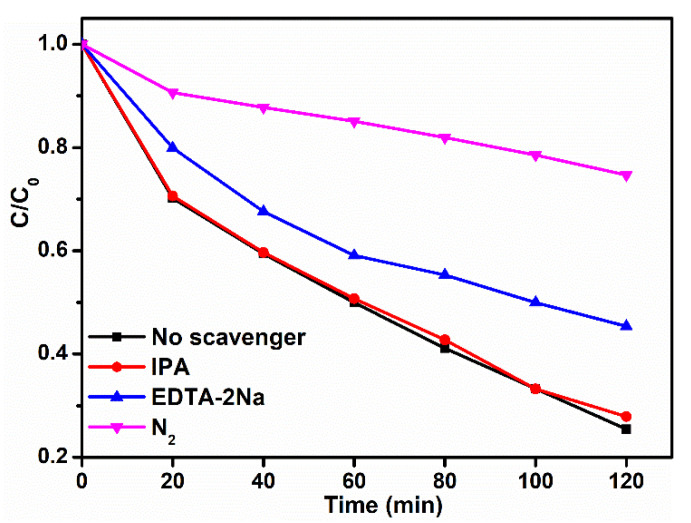
Reactive species trapping experiments over CISCN-2.

It is worth studying the possible reaction mechanism during the TCH degradation. In general, to distinguish the main active species generated in the photocatalytic reaction process, active species trapping experiments were conducted. Isopropyl alcohol (IPA) and disodium ethylenediaminetetraacetate (EDTA-2Na) were chosen as scavengers for removing •OH and h^+^, respectively. N_2_ was continuously bubbled into the reaction system for removing the soluble oxygen, eliminating the formation of •O_2_^−^ [54,55]. As shown in Figure 8, the addition of IPA (1 mM) had an extremely small effect on the photocatalytic performance of the CISCN-2, indicating that the role of •OH formed during the photocatalytic process could be ignored. After the EDTA-2Na (1 mM) was introduced, an apparent decrease in the TCH removal ratio could be observed; it was decreased from 74.6% to 54.6%, implying the photoexcited holes made contributions to the degradation of TCH. Meanwhile, when N_2_ was continuously bubbled into the reaction system during the whole photocatalytic TCH degradation process, a dramatic decrease could be observed; the removal ratio of TCH was decreased from 74.6% to 25.3%, implying that •O_2_^−^ played the prominent role in TCH removal. Therefore, the generated •O_2_^−^ played a predominant role in the TCH photodegradation procedure, but in the meantime, the contributions of photoexcited holes also could not be overlooked.

**Figure 9:**
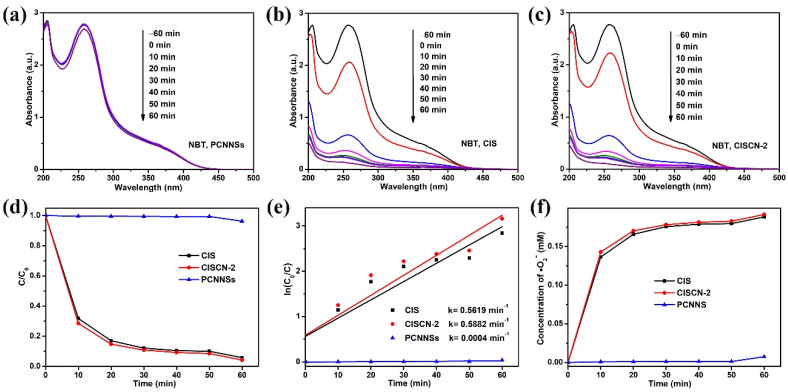
Time courses of absorption variation of NBT over (**a**) PCNNSs, (**b**) CIS, and (**c**) CISCN-2. (**d**) Time-dependent photodegradation plots of NBT over different catalysts; (**e**) the corresponding pseudo-first-order kinetic fitting curves, (**f**) time-dependent concentration curves of •O_2_^−^.

The generation of •O_2_^−^ was measured by the NBT transformation experiment. Because the reaction between •O_2_^−^ and NBT occurred at a mole ratio of 4:1, the content of the •O_2_^−^ could be estimated by the change in NBT. Figure 9a–c were the typical time courses of absorption variation of NBT over the PCNNSs, CIS, and CISCN-2. For PCNNSs, a delayed change could be observed. However, the intensity of the typical absorbance signal at 260 nm for NBT exhibited a noteworthy decrease over CIS and CISCN-2, indicating the generation of •O_2_^−^ during the photocatalytic reaction. Figure 9d,e displays the change in NBT concentration and the corresponding kinetic fitting curves. Obviously, the reaction between •O_2_^−^ and NBT over the CISCN-2 had the fastest rate. Meanwhile, the •O_2_^−^ generated over CISCN-2 was the largest amount produced among these three samples (Figure 9f), which further verified the enhancement of the photocatalytic activity over CISCN-2 in •O_2_^−^ production.

**Figure 10:**
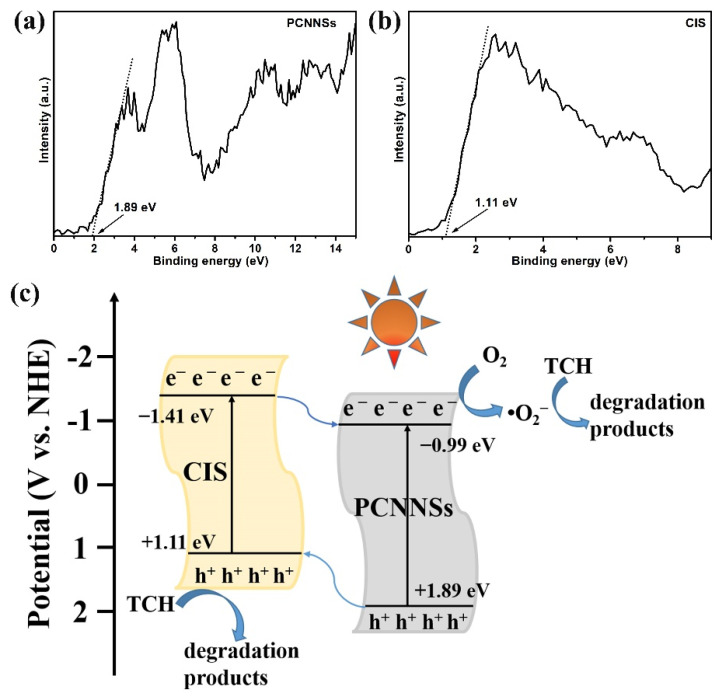
(**a**) VB-XPS of PCNNSs, (**b**) VB-XPS of CIS, and (**c**) schematic diagram of photogenerated charge transfer pathway and possible photocatalytic mechanism in CdIn_2_S_4_/g-C_3_N_4_ heterojunction toward TCH degradation.

Moreover, measurements of the band edge positions of the single component were necessary, as they could further verify the possible photocatalytic mechanism. Figure 10a, b exhibits the corresponding VB-XPS spectra of the PCNNSs and CIS. It can be clearly observed that the valence band (VB) position of PCNNSs and CIS was +1.89 and +1.11 eV, respectively. Therefore, based on the evaluation with UV-vis DRS, the conduction band (CB) position of PCNNSs and CIS could be determined by the equation E_g_ = |E_CB_ − E_VB_| to be −0.99 and −1.41 eV, respectively. The possible photocatalytic mechanism and the transfer path of photogenerated charge carriers were proposed on the basis of active species trapping experiments and the band edge positions of the PCNNSs and CIS, as displayed in Figure 10c. When the photocatalytic reaction system was exposed to visible light (λ > 400 nm), both PCNNSs and CIS could be excited to generate electron-hole pairs. Owing to the more negative CB position of CIS than that of PCNNSs, the photoexcited electrons on the CB of CIS would transfer toward the CB of PCNNSs, which could react with soluble oxygen molecules, leading to the formation of •O_2_^−^ with strong oxidation ability that could oxidize organic pollutants. Simultaneously, the photoinduced holes on the VB of PCNNSs with more positive potentials would migrate to the VB of CIS; the photoinduced holes could directly oxidize organic pollutants. This spatial separation of photoinduced charge carriers might be one of the reasons for the enhanced photocatalytic performance.

## 4. Conclusions

In summary, 2D/2D CdIn_2_S_4_/g-C_3_N_4_ heterojunctions were productively constructed through a facile electrostatic self-assembled route. The unique 2D/2D nanostructures exhibited tight interfacial contact and a large contact area, which could provide more channels for the transfer of photoinduced charge carriers and shorten the transfer distance of the charge carriers, realizing a higher separation efficacy of photoexcited electron-hole pairs, thus benefiting molecular oxygen activation. The optimized 2D/2D CdIn_2_S_4_/g-C_3_N_4_ heterojunction exhibited the highest photocatalytic performance and photostability toward TCH degradation. The improved photocatalytic activity could be attributed to the high separation and transfer efficacy of the photoexcited charge carriers, achieving a higher molecular oxygen activation efficiency.

## Figures and Tables

**Table 1:** Degradation efficiency (DE) of TCH over previous reports and CISCN-2 in this work.

Sample	TC (mg/L)	Dosage (g/L)	t (min)	Light Source	DE (%)	Refs.
Co/V-g-C_3_N_4_	10	0.5	120	250 W Xe lamp	64.3	[5]
2D/3D g-C_3_N_4_	10	0.5	120	250 W Xe lamp	69.6	[6]
Nitrogen-deficient tubular g-C_3_N_4_	10	1.0	150	300 W Xe lamp	84.3	[7]
BN QDs/g-C_3_N_4_	10	1.0	60	300 W Xe lamp	82	[8]
WO_3_/g-C_3_N_4_	25	0.5	120	300 W Xe lamp	70	[28]
Bi/α-Bi_2_O_3_/g-C_3_N_4_	10	1.0	180	300 W Xe lamp	91.2	[29]
Nb_2_O_5_/g-C_3_N_4_	10	0.5	150	250 W Xe lamp	76.2	[30]
CdIn_2_S_4_/g-C_3_N_4_	50	0.6	120	300 W Xe lamp	83.6	This work

## Data Availability

Data supporting this study are available within the article.
